# A cortical thinning signature to identify World Trade Center responders with possible dementia

**DOI:** 10.1016/j.ibmed.2021.100032

**Published:** 2021-04-22

**Authors:** Sean A.P. Clouston, Minos Kritikos, Yael Deri, Megan Horton, Alison C. Pellecchia, Stephanie Santiago-Michels, Melissa A. Carr, Sam Gandy, Mary Sano, Evelyn J. Bromet, Roberto G. Lucchini, Benjamin J. Luft

**Affiliations:** a Program in Public Health and Department of Family, Population, and Preventive Medicine, Renaissance School of Medicine at Stony Brook University, Stony Brook, NY, 11794, USA; b Department of Environmental Medicine and Public Health, Icahn School of Medicine at Mount Sinai, New York, NY, 10029, USA; c Stony Brook World Trade Center Wellness Program, Renaissance School of Medicine at Stony Brook University, Stony Brook, NY, 11794, USA; d Center for Cognitive Health and NFL Neurological Care, Department of Neurology, Icahn School of Medicine at Mount Sinai, New York, NY, 10029, USA; e Department of Psychiatry and Mount Sinai Alzheimer’s Disease Research Center, Icahn School of Medicine at Mount Sinai, New York, NY, 10029, USA; f Department of Psychiatry, Renaissance School of Medicine at Stony Brook University, Stony Brook, NY, 11794, USA; g Department of Medicine, Renaissance School of Medicine at Stony Brook University, Stony Brook, NY, 11794, USA

**Keywords:** Cognitive impairment, Cortical thickness, World trade center responders

## Abstract

**Introduction:**

World Trade Center (WTC) responders have a high risk of early-onset cognitive impairment (CI), but little is known about the etiology including the extent to which CI in WTC responders is accompanied by cortical atrophy as is common in progressive diseases causing age-related CI such as Alzheimer’s disease and related dementias. In the current study, we entrained an artificial neural network (ANN) to determine the accuracy of cortical thickness (CTX) on magnetic resonance imaging to identify World Trade Center responders at midlife (aged 44–65 years) with possible dementia.

**Methods:**

A total of 119 WTC responders (57 with CI and 62 with intact cognition) underwent a structural MRI scanning protocol including T1-weighted MPRAGE as part of two imaging studies. The discovery study was divided into training and validation samples, while a second replication sample was used. An ANN was trained using regional CTX measured across 34 unilateral regions of interest (ROIs) using Freesurfer software and ‘Desikan-Killiany’ brain atlas. The discovery sample was used for model development, and the replication sample was used to evaluate predictive accuracy.

**Results:**

In the WTC responder cohort, the ANN algorithm showed high discrimination performance for CI. The ANN model using regional CTX data from both hemispheres achieved an area under the receiver operating characteristic curve (AUC) of 0.96 95% C.I. = [0.91–1.00] (Accuracy = 96.0%, Precision = 97.8%, Recall = 95.8%, Sensitivity = 95.8%, Specificity = 98.0%, F1 = 96.8%) for the discovery sample and AUC = 0.90 [0.70–1.00] (Accuracy = 90.0%, Precision = 90.0%, Sensitivity = 90.0%, Specificity = 90.0%, F1 = 90.0%) in the replication sample.

**Conclusion:**

Analysis of bilateral regional CTX data derived from T1-weighted MPRAGE images by ANN analysis demonstrated excellent accuracy in distinguishing WTC responders with early-onset CI.

## Introduction

On September 11th, 2001, hundreds of thousands of people including those residing in the Greater New York area, watched first hand as the World Trade Center (WTC) collapsed after two planes were flown into the towers. On that day and in the months thereafter, tens of thousands of men and women worked in search, rescue, and recovery operations (hereafter named “Wresponders”). While studies have documented the extreme conditions to which WTC responders were exposed on November 09, 2001 as well as the chronically elevated risk of psychiatric illness among these men and women responders [1], little is known about reasons for increased risk of neurological diseases in this cohort. However, earlier studies have indicated that post-traumatic stress disorder (PTSD) among veterans of the Vietnam, Iraq, and Afghanistan wars can be associated with cognitive impairment (CI) and cortical atrophy [2,3]. Other studies have suggested that severe and/or chronic exposure to inhaled nano-sized particulate matter (PM < 2.5 μm [PM2.5]) have been linked to amyloidogenesis in mice [4], CI in older women [5], and may cause neurodegeneration [6]. WTC responders were also exposed to inhalation of elevated levels of PM2.5 [7].

Prior efforts to identify biomarkers for CI have determined that the topography of cortical atrophy can help to determine and differentiate subtypes of dementia [8,9]. For example, cortical thickness (CTX), as measured using magnetic resonance imaging (MRI), reliably quantifies neurodegeneration in mild cognitive impairment [10] and diagnosed Alzheimer’s disease (AD) [11], with focal reductions evident in the medial temporal lobe and the posterior cingulate [12]. Despite their relative youth, ongoing research has detailed that WTC responders are at increased risk for aging-related CI [13]. Yet, whereas biomarkers for Alzheimer’s disease are increasingly well understood [14], the topography of cortical atrophy among WTC responders does not match known Alzheimer’s disease topographical signatures [15], and while predictive the AD fingerprint has relatively low accuracy for detecting CI in this population (AUC 0.68 [0.57–0.78]) [16].

CI is most severe when accompanied by cortical atrophy [17,18] yet while research in WTC responders suggests that cortical atrophy is present [15], the commonality of cortical atrophy in WTC responders with CI remains unclear. Nothing is known about the accuracy of CTX measurement to accurately discriminate CI in WTC responders at midlife. The present study filled these gaps in three ways: first, since exposures at the WTC disaster site were severe and unique this study sought to better understand the severity of CI among WTC responders by going beyond reliance on AD signatures to characterize more completely the biomarker signature associated with CI among WTC responders; second, it created a novel artificial neural network (ANN) algorithm to reliably identify responders with CI and reported classification accuracy of neuroimaging parameters as a main outcome; and finally, we uniquely examined cortical atrophy in responders with MCI and dementia to determine reliable and consistent regional distribution of cortical atrophy.

## Methods

### Participants

The present study utilized results from two imaging studies investigating WTC responders with CI as compared to WTC responders who were cognitively unimpaired. In both cases, participants were purposively recruited from a single clinic-based monitoring program in the WTC Health Program [19] whose participants additionally participated in serial administration of the Montreal Cognitive Assessment (MoCA) [20]. Eligible participants completed neuroimaging if they consented and did not drop out during the screening or scanning visits. Participant groups in both studies included WTC responders with CI, and those determined to be cognitively unimpaired using a standard diagnostic protocol as outlined below. By design, cases were matched to controls using demographic characteristics including on age, sex, occupation, education, posttraumatic stress disorder, and minority status. Women and responders from minority backgrounds were over-sampled in this study to improve generalizability. The sample of the first study (N = 99) served as the *discovery sample* (48 medically healthy individuals with dementia, 51 demographically matched cognitively unimpaired controls) while the *replication sample* included ten medically healthy WTC responders presenting with mild cognitive impairment (MCI) and ten demographically matched cognitively unimpaired controls.

*Eligibility criteria* for both studies were ages 44–65, fluent in English, and completion of a diagnostic assessment of WTC-related PTSD. Cognitive status was confirmed at screening visits.

*Exclusion criteria* for both studies included having a body mass ≥40, history of psychosis, history of diagnosed neurological conditions including diagnosed Alzheimer’s disease, other dementias, major stroke, multiple sclerosis, and Parkinson’s disease, severe head trauma from the WTC or a history of head trauma, current liver disease, and current use of cognitively active medications. Subjects also satisfied eligibility criteria for MRI scanning including no known claustrophobia, and no known metal implants or shrapnel that was not deemed MRI-safe.

### Ethics

The Institutional Review Boards at both Stony Brook University and the Icahn School of Medicine at Mount Sinai approved study procedures. Participants provided written informed consent to participate in all research studies.

### Measures

The following demographic characteristics were recorded: age, sex (female *versus* male), occupation (law enforcement *versus* other), and educational attainment (university degree *versus* at least some college *versus* high school). Upon enrollment, all eligible responders were screened to provide more detailed information about everyday functioning and to ensure case status. In both studies, responders’ age, sex, and race/ethnicity as well as occupation and education were matched across cognitive case groupings.

### Image acquisition

In the *discovery sample,* three-dimensional T1-weighted magnetization-prepared rapid gradient echo (T1-MPRAGE) (TR = 1900 s, TE = 2.49 ms, TI = 900 ms, Flip Angle = 9°, acquisition matrix: 256 × 256 and voxel resolution: 0.89 × 0.89 × 0.89 mm) were obtained on a 3 T S Biograph mMR. For incidental pathology screening, T2-weighted anatomical scans of the whole brain using a turbo spin-echo pulse sequence (34 axial slices, TR = 6170s, TE = 96 ms, Flip angle = 150°, acquisition matrix = 320 × 320, voxel size = 0.36 × 0.36 3 mm) were also acquired. While being collected on the same model of scanner, the *replication sample* had slightly different acquisition parameters for T1-MPRAGE (TR = 2300 s, TE = 3.24 ms, TI = 900 ms, Flip Angle = 9°, acquisition matrix: 256 × 256 and voxel resolution: 0.87 × 0.87 × 0.87 mm).

### Image processing

T1-MPRAGE images were used to obtain cortical thickness measures utilizing the standard, automated cortical reconstruction pipeline of FreeSurfer V.5.3 as described in previous publications [21,22]. Briefly, the surface models were inflated and registered to a standard spherical surface atlas before being smoothed and recorded [23].

### Measures

#### Cortical Thickness:

CTX is a consistent measure of brain atrophy that is commonly used in studies of AD and other related dementias [24]. We focused on CTX both because the validation of these features indicates that CTX is preferable to other possible features because it is highly sensitive to neurodegeneration but less sensitive to unmeasured confounding, and because performing inference on the raw images directly was significantly more computationally expensive than was feasible for the investigators to undertake without specialized hardware. Indeed, CTX compares favorably with gray matter volume, whole-brain volume, and hippocampal volumes because, while all of these measures can be indicators of neurodegeneration, CTX can be quantified across multiple brain regions, and is generally thought to be minimally related to intra-cranial volume and sex [25]. CTX measurements were obtained by calculating the mean distance between gray and white matter boundaries and the outer pial surface of the cerebral cortex. Regional CTX was calculated in each hemisphere separately for the 34 subregions defined by the Desikan-Killiany atlas [23]. Unilateral and bilateral CTX estimates were recorded.

#### Diagnosis of Cognitive Impairment:

In the discovery sample we relied on data from 48 individuals with mild dementia as diagnosed using NIA-AA standards as indicated by medically healthy individuals with CI identified using a standard cutoff with evidence of functional limitations consistent with possible dementia [26]. Global cognitive functioning wqs measured using the Montreal Cognitive Assessment (MoCA), a widely used measure of cognitive functioning with a clinical application and was developed to objectively and reliably identify age-related CI [20].In the *discovery sample*, possible mild dementia was characterized by evidence of cognitive impairment (MoCA≤20) without underlying medical complications. To match these patients, data from 51 cognitively unimpaired WTC responders (MoCA≥26) as controls. The *replication sample* included ten medically healthy WTC responders presenting with mild cognitive impairment (MCI), as defined by the observed onset of mild CI (MoCA≤23) coupled with evidence of cognitive decline but without functional limitations [27], and ten cognitively stable WTC responders served as controls (two observations >12 months apart with MoCA≥26).

The discrepancy between the *discovery sample* with CI versus the *replication sample* with mild CI arose primarily because these two studies originally addressed different levels of CI within the same cohort of individuals. Since the present study required a *replication sample* to validate the predictive power of the ANN with the *discovery sample* from within the same cohort, these two studies were a good match to fulfill that requirement.

*Computer-Assessed Cognitive Performance* (CogState) was measured utilizing a brief, 20-min computer-administered approach [28] that uniformly measures fluid cognition using data and metadata during a game-like task. Cognitive domains measured included: reaction speed, processing speed, cognitive efficiency, intra-item response variability, attention, executive function, visual memory, paired associate learning, visuospatial learning and memory, and working memory. Validity checks are built into the scoring methodology. Prior work with CogState in this population has found that cognitive dysfunction in several cognitive domains such as reaction speed, processing speed, and memory was associated with both long-term exposures to the WTC sites and with symptoms consistent with severe and chronic PTSD [29].

### Statistical analyses

**Fig. 1.**
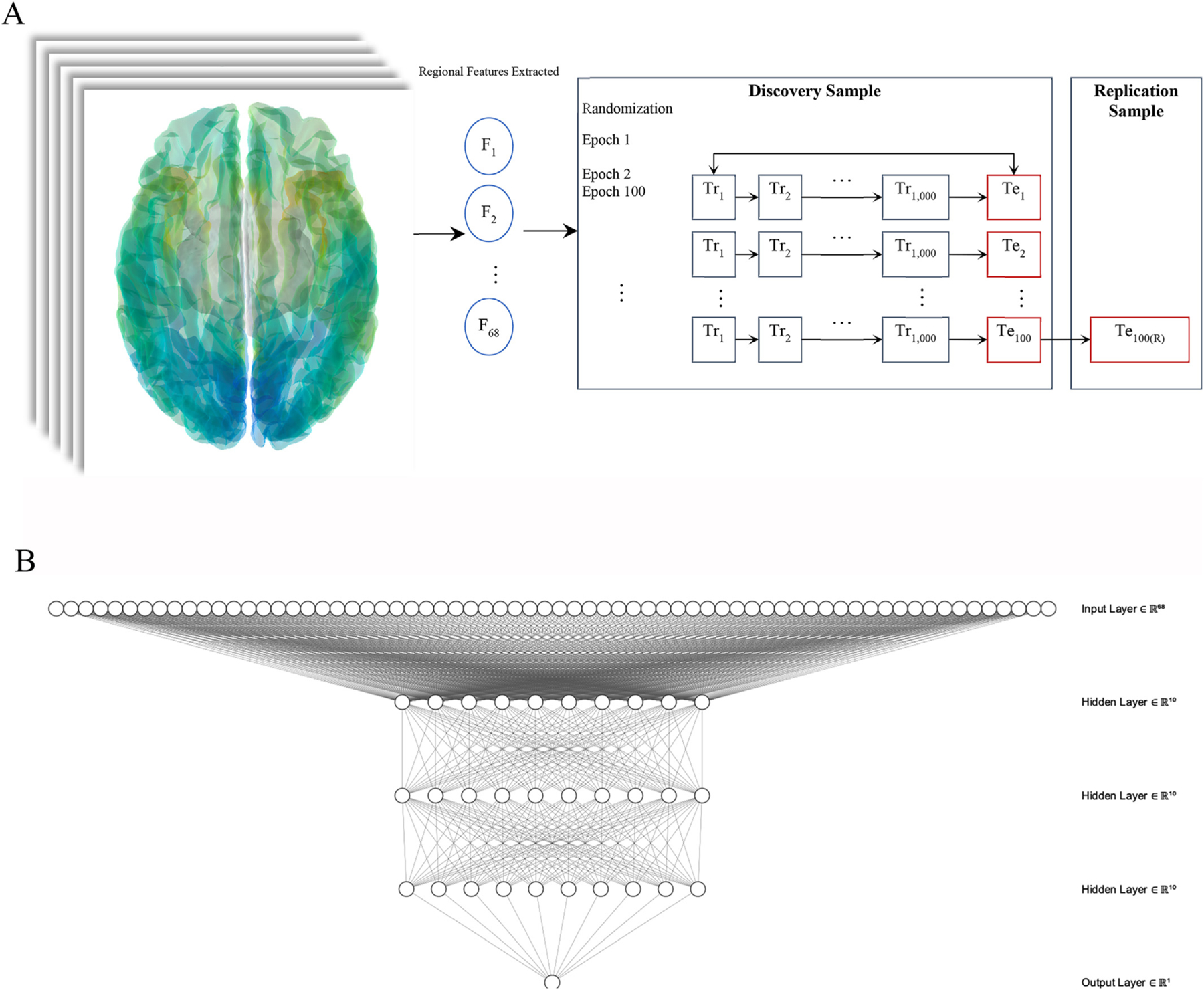
Artificial neural network structure and training protocol **Panel A:** Workflow for the training and testing process used to train and validate the artificial neural network. The figure notes training efforts (Tr_i_) leading into initial testing circumstances (Te_i_), and finally upon training/testing conclusions on replication (Te_i(R)_), where “i” indicates training epoch. **Panel B:** Architectural diagram for the artificial neural network using three hidden layers with ten nodes each incorporating 68 bilateral measures of regional cortical thickness.

Descriptive characteristics were provided using mean and standard deviations, or frequencies and percentages where noted. In this study, confounding from central variables including age was completed by the use of matching in the design phase. The following describes how we trained, tested, and then examined out of sample replicability for our artificial neural network. Fig. 1 provides the ANN architectural diagram (panel A) and, in panel B, the training, testing, and replication protocol.

As seen in Fig. 1A, the analytic plan focused on studying the accuracy of mean CTX within each cortical sub-region before examining mean hemispheric CTX. Next, we applied the learning ANN to identify differences between brain subregions [30]. To accomplish this task, we completed all of the training and validation efforts in the discovery sample before then, as a final stage, applying the scoring to the replication sample. We relied on randomized K-fold cross-validation to create the learning process – because training requires more statistical power than does validation, we apportioned 66% of discovery sample cases to training and 33% were retained for intra-discovery validation to determine whether the ANN was learning. Learning curves were reported using this training session.

The ANN incorporated (Fig. 1B for architecture) bilateral brain regions from both hemispheres as input layers (spread = 0.5). The ANN had three hidden layers with ten nodes in each layer and out output layer, which recorded the cortical atrophy risk score. ANN training was accomplished in two steps. First, in the training subsample, we defined a training session that trained the ANN to identify the outcome and each training session randomly sorted the cases and controls within the dataset to ensure that case order was not identified by the ANN as a model parameter. The learning rate, a model parameter used to tune the learning rate *versus* computational speed tradeoff, was set to η = 0.10. Second, epochs were defined by randomly selecting cases from the discovery sample to create a new training session and retrained the ANN using the new subset of cases. At each epoch, the area under the receiver operating curve (AUC) with corresponding 95% confidence intervals [95% C.I.] was reported as the primary measure of model performance for both the training and validation samples. There were 100 epochs each of which incorporated 1000 making a total of 100,000 training opportunities for the ANN in this analysis. Learning curves were created using a moving average of the AUC reported in each epoch and mean within-epoch AUCs and 95% confidence intervals were provided.

The final ANN reported marginal signal intensity estimates arrays to provide a sense of specific regional findings. Marginal signal intensity estimates for each ROI used by the ANN to show signal intensity. Signal intensity provides a standardized metric indicating the difference in output estimates between the ANN with and without the input variable of interest, in this case the specific region of interest. Finally, after the ANN was completely trained, the AUC was reported both in the whole *discovery (n = 99)* and *replication (n = 20) samples*. Since cutoffs can help differentiate individuals with high versus low risk of CI; Youden’s method was used in the replication sample to determine conservative cutoffs for the best performing model and these cutoffs were used to categorize outcomes in both samples and Youden’s index (J) was reported [31]. At this time, we also reported the classification ANN’s AUC along with accuracy, precision, specificity, sensitivity, F1 score, positive likelihood ratio (LR+), and negative likelihood ratio (LR−). Analyses were preformed using Stata 16/SE [StataCorp].

## Results

Table 1 shows the relevant characteristics of WTC responders by cognitive impairment status. On average, WTC responders were in their mid-fifties and were predominantly male.

Examining the accuracy of the predictive ANN revealed that any particular mean bilateral CTX was, on its own, a weak to moderate indicator of CI (Table 2). Mean CTX of the unilateral whole hemisphere and also unilateral single-region information demonstrated weak to moderate predictive power on their own in the *replication sample* with the strongest regional predictive value located in the right and left lateral occipital cortices (AUC = 0.72 [0.44–1.00] and 0.75 [0.52–0.99] respectively), right postcentral gyrus (AUC = 0.74 [0.50–0.98]) and right rostral anterior cingulate cortex (AUC = 0.78 [0.55–1.00]).

The ANN became, during the training and validation process, increasingly accurate at separating responders with WTC-CI (learning curve provided in Appendix Fig. 1). The final output was a score showing preeminent performance in both *discovery* and *replication samples* (Table 2) that generated a risk score with excellent AUC in the *replication sample*. For comparison, we provided overall fit metrics for each region of interest in both samples and also across the entire sample using the optimized cut-point shown.

**Fig. 2.**
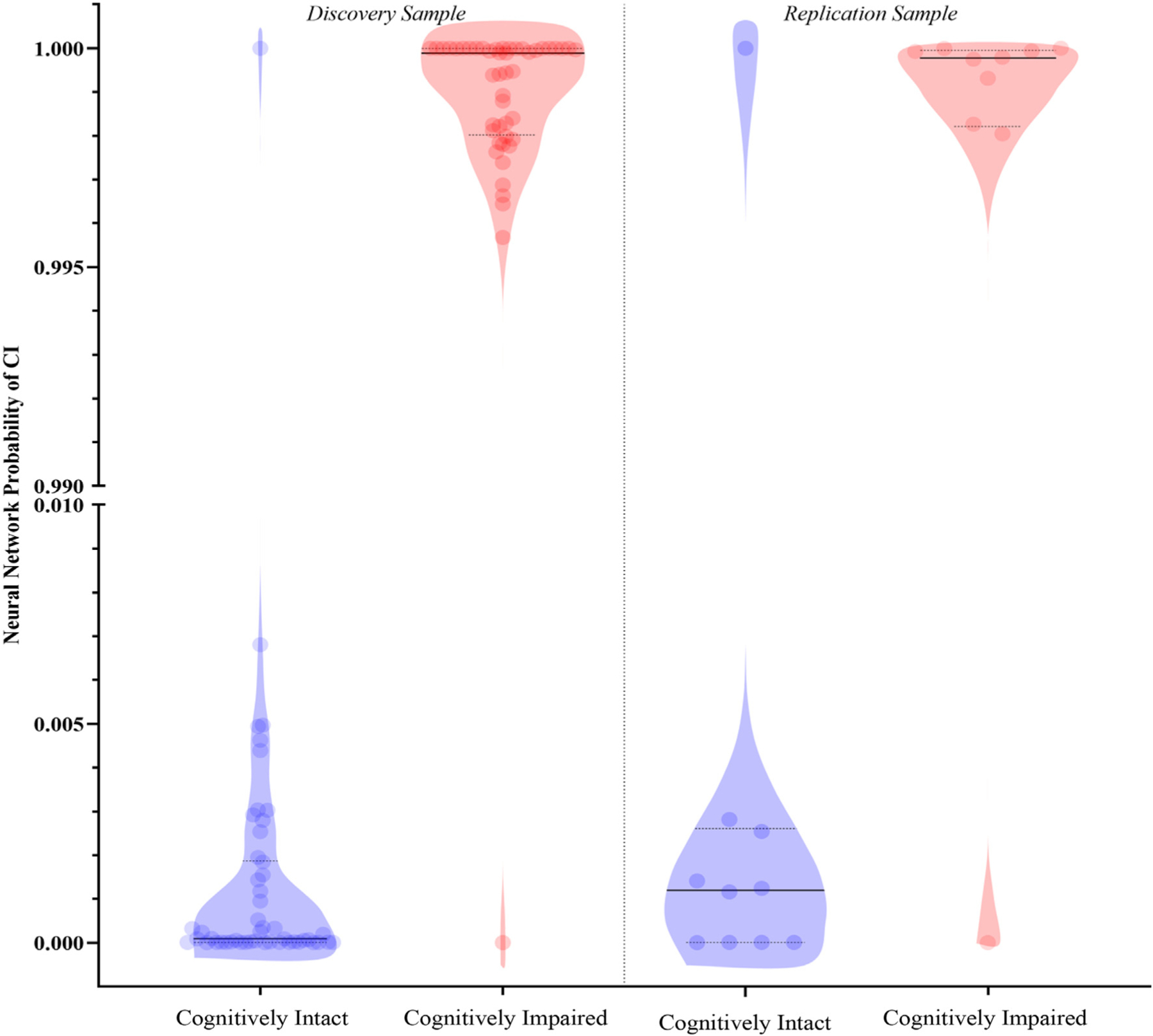
Violin plots with showing the distribution of artificial neural network scores for participants classified as cognitively unimpaired (blue) and cognitively impaired (red). The score for each participant is shown using a translucent dot, while the median and interquartile range are shown using solid and dotted black lines respectively.

Examining the distribution of the ANN-derived risk score (Fig. 2), showed that groups appeared highly separated between CI and unimpaired groups [the trained ANN is provided in Appendix Model 1].

Cortical regions comprising the risk score were determined from the signal matrix (provided in Table 3) shows an overall indication of how important each region was to the ANN scoring. Regions with non-zero scores in Table 3 suggest that, after training, the ANN algorithm learned to reliably distinguish between WTC responders with and without CI using CTX signature of decay as indicated by marginal estimates. Unilateral regions displayed prominent contributions to the discrimination process including, for example, the right cuneus, right middle temporal, rigght pericalcarine, right precentral, and right superior frontal regions.

**Fig. 3.**
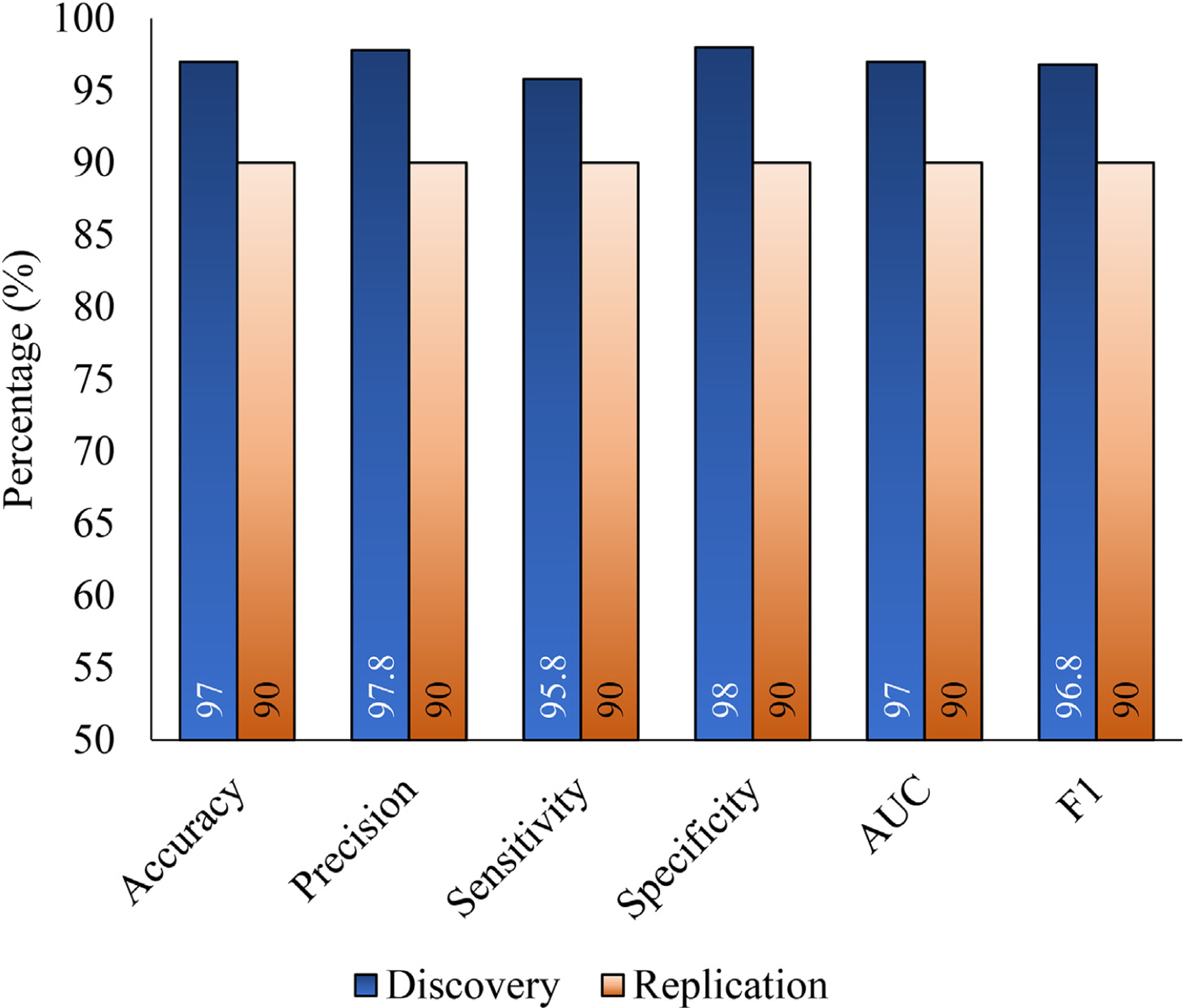
Bar charts showing estimated measures of accuracy in the discovery (blue bars) and replication (orange bars) samples. Higher overall accuracy is indicated as proximity to the ceiling (100%), while poorer performing metrics would approach a floor of 50% because all scores were oriented in the same direction. AUC indicates the area under the receiver-operating curve.

Cut-point detection efforts were based on the risk score estimated by the ANN suggested that the optimal *cut-off score* in the discovery sample was 0.50 (AUC = 0.96, 95% C.I. = [0.91–1.00]; J = 0.77; LR+ = 48.9; LR− = 0.04). As is evident in Fig. 3, at this cut-off 46/48 (91.7%) of responders with CI were correctly classified, while 50/51 (98.0%) of cognitively unimpaired responders successfully classified as unimpaired in the *discovery sample*. In the *replication sample*, 9/10 (90%) responders with MCI were accurately classified and 9/10 (90%) cognitively unimpaired responders were correctly classified (AUC = 0.90 [0.70–1.00], LR+ = 9.0, LR− = 0.11).

Our ANN incorrectly identified three responders incorrectly classified as cognitively unimpaired (false negative – type II error), while two cognitively unimpaired responders were incorrectly identified as CI (false positive – type I error). Inspecting the data from the two type II errors revealed that when compared to the WTC responders appropriately identified as CU, these three displayed higher mean unilateral CTX in the right (2.42 *versus* 2.36, difference = 0.06 mm) and left hemispheres (2.43 *versus* 2.37, difference = 0.06 mm) respectively. When comparing the two cases of type I error to WTC responders identified as CU, these participants displayed a lower mean hemispheric CTX in the right (2.34 *versus* 2.44, difference = ‒0.10 mm) and left hemispheres (2.45 *versus* 2.36, difference = ‒0.09 mm) respectively.

In exploratory analyses, we further investigated the association between neural network risk scores and performances in a range of cognitive domains as assessed on a computer-assisted cognitive exam that was not employed in the characterization of CI or cognitively unimpaired in either the *discovery* or *replication sample*. The ANN score was associated with cognitive performances across a range of cognitive domains in the expected directions (Appendix Table 1).

## Discussion

As of 2020, 79,189 responders were documented to have worked on response efforts at the WTC [32]. These responders were exposed to potentially injurious events, with many experiencing PTSD or early-onset CI. To date, little is known about the etiology for why early signs of cognitive decline might be present. Recent work has begun to identify signs of neurodegeneration in WTC responders with cognitive impairment [15,43,44]; however, no prior work has determined the accuracy of cortical atrophy to identify CI in WTC responders. Identifying accurate methods for identifying patients is critical to diagnosis and to monitoring patient symptomatology. This is the first study to apply a neural networks approach to characterize regional cortical atrophy in WTC responders. This is also the first study to compare responders with mild cognitive impairment with those with dementia, and to note that those with MCI may be biologically similar to those with dementia indicating the potential for progressive disease. As such, we deemed it important to develop a useable signature by using cortical thickness as a utility that could allow us to reliably identify which responders are at risk of WTC-CI. The resulting efforts suggested that the signature identified here was able to reliably differentiate between CI and cognitively unimpaired in WTC responders. Furthermore, this signature could allow us to identify which WTC responders might be at the highest risk for developing WTC-related CI. Notably, the cortical atrophy signature offered a high degree of accuracy in both the training and for prediction in a second, unrelated, study with different imaging parameters. These results are promising in their ability to reliably identify responders with mild cognitive impairment [33].

### Regionally specific cortical thinning in WTC-CI

Prior research has suggested that cognitive impairment most often results from neurodegenerative diseases such as AD [34], as well as other related disorders such as frontotemporal lobular degeneration (FTD) [35], progressive supranuclear palsy (PSP) [36], Parkinson’s disease (PD) [37], dementia with Lewy bodies (DLB) [38], and amyotrophic lateral sclerosis (ALS) [39]. These studies suggest that the areas identified in the derived WTC-CI signature may have identified regions active across a range of neurodegenerative conditions including, for example, regions within the temporal lobe that are commonly implicated in AD with focal points in the medial temporal, inferior temporal, temporal pole as well as subregions of the frontal lobe, the right parietal lobe as seen in PD, and the left frontal lobe and supramarginal regions as seen in PSP.

Our findings partially overlapped with five of nine brain regions classically associated with the AD-related CTX signature including the pericalcarine cortex, inferior temporal gyrus, superior frontal gyrus, and rostral medial frontal cortex [40]. Indeed, application of the AD fingerprint resulted in relatively low accuracy in this population. Thus, in the anatomical underpinnings of WTC-CI we found some overlap with the cortical atrophy characteristics of AD. Yet, WTC responders also manifested cortical atrophy in non-AD-related regions including in the right frontal pole, precentral, lateral orbitofrontal, and left insula. Given the similarity in brain regions impacted by diseases with very different etiologies and symptoms, these data may be most useful in providing clues as to the etiology of the disease and about the anatomical basis of WTC-related cognitive pathology. For example, changes in the lateral orbitofrontal cortex have been linked to impairments in learning-based decision-making [41,42]. Further work will be required to determine whether a clinicopathological correlation exists in WTC-CI in the same way that it does in other memory-impairing neurodegenerative diseases including, for example, AD, FTD, PD, and PSP.

The ANN regional effect sizes seem to suggest differences in spatial location of cortical thinning between hemispheres pointing towards a laterality effect. These findings, if confirmed, would not be consistent with the CTX signature typical for AD[40]. One explanation for this discrepancy could be that indeed, unlike in AD, WTC responders with CI display more laterality in cortical atrophy. An alternative explanation may be that the bilateral regions have the same contribution and therefore the model only needed to use to account for the contribution from both sides.

### Strength and limitations

This study used an ANN teaching and learning platform to develop an accurate objective biological indicator of CI in WTC responders. While this is a pivotal strength in this study, there are notable weaknesses including, for example, the reliance of the analysis on measurements from two separate and relatively small imaging studies despite being recruited from a single WTC epidemiologic cohort, without the use of an external control database, though using an identical pipeline to analyze the data. Given these necessary forces, we tried to improve replicability by using validation on a different dataset derived from a different study with individuals defined using different diagnostic criteria to identify possible mild dementia and mild cognitive impairment, with MRI data collected in a different imaging suite and using different acquisition parameters. While this is in some senses a limitation, it may also be seen as a strength since these types of dis-similarities allowed our practice to imitate some of the most common differences experienced when comparing similar individuals across imaging sites and diagnostic protocols and, therefore, reproduces a practical replication effort. However, these also represent sources of variability that may reduce our ability to generalize and induce variation that could bias results. Another limitation is that the ANN learning module independently chose the final results that were presented. This study did not incorporate additional measures of cortical health including cortical density and complexity. To that end, we might also suggest that future work might usefully refine the ANN to improve reliability to differentiate responders at risk of CI. Additionally, while the region-based analyses allowed us to apply an external ANN to the process being used in this study, it is worth noting that future research should refine this protocol by implementing it within an environment that allows for vertex-wise analyses and by using longitudinal data to determine the rate of progression of CTX atrophy in WTC responders. Furthermore, there is a need to replicate this work to determine whether others might benefit from the application of the ANN to cortical atrophy measured in other traumatized populations to determine whether it yields similar predictive accuracy for detecting early-onset CI. Finally, while we oversampled women and visible minorities in this study, the population characteristics make it somewhat difficult to assess levels of bias in this study making replication in studies with more women and people from minority backgrounds is necessary to quantify the potential for bias in this study.

## Conclusion

WTC responders experienced a severe and unique exposure, with both psychological trauma and the potential for inhalation of neurotoxins. Prior work has detailed symptoms consistent with this event, while further efforts have suggested that signs of neurodegeneration in the cortical gray matter is evident, but that patterns of neurodegeneration do not necessarily match those of other diseases. As such, this paper fills a particular need for this population to derive biomarkers for WTC-related CI. However, this paper also implemented a novel region-based technique for reliably identifying neurodegenerative disease in highly exposed WTC responders. In so doing, this study provided the basic characterization needed to begin to generate a new subtype of AD or a related dementias and/or to identify a novel neurodegenerative disorder at midlife.

## Supplementary Material

Appendix Tables and Figures

Supplemental Appendix Code

## Figures and Tables

**Table 1:** Sample characteristics for the discovery and replication samples.

Characteristic	Discovery sample		Replication Sample	
	Cognitively unimpaired (N = 51)	Cognitively Impaired (N = 48)	Cognitively unimpaired (N = 10)	Cognitively Impaired (N = 10)
Age	56.40 ± 4.50	56.30 ± 5.90	55.70 ± 4.40	56.3 ± 5.3
Sex				
Male	80.8%	76.6%	90.0%	90.0%
Female	19.2%	23.4%	10.0%	10.0%
Occupation				
Law enforcement	80.8%	66.0%	40.0%	70.0%
Other	19.2%	34.0%	60.0%	30.0%
Education				
High school or less	17.3%	29.8%	0.0%	0.0%
Some College	50.0%	46.8%	80.0%	60.0%
University Degree	32.7%	23.4%	20.0%	40.0%
Cognitive Domains				
Paired-Associate Learning	0.70 ± 0.19	0.56 ± 0.10***	0.65 ± 0.21	0.59 ± 0.09
Item Response Variability	0.09 ± 0.03	0.12 ± 0.04***	0.10 ± 0.04	0.09 ± 0.03
Reaction Speed	7.90 ± 0.70	7.20 ± 0.90***	8.10 ± 0.50	7.40 ± 0.70*
Spatial Learning	57.20 ± 15.30	95.60 ± 61.80***	64.10 ± 18.90	77.60 ± 28.50
Spatial Memory	9.40 ± 4.30	17.70 ± 11.10***	13.50 ± 6.70	15.30 ± 7.20
Processing Speed	6.50 ± 0.40	6.10 ± 0.60***	6.50 ± 0.40	6.40 ± 0.60
Attention	1.42 ± 0.14	1.28 ± 0.22***	1.39 ± 0.18	1.41 ± 0.20
Visual Memory	0.99 ± 0.12	0.91 ± 0.10***	1.04 ± 0.10	0.94 ± 0.06**
Throughput	0.33 ± 0.04	0.30 ± 0.03***	0.35 ± 0.03	0.31 ± 0.02**

**Note:** Mean ± Standard deviations or percentages are presented for continuous and categorical variables respectively. P-values examined within-sample differences between CI and non-CI individuals and were derived from t-tests for continuous variables, and χ^2^ tests for categorical variables:

* p < 0.05

** p < 0.01

*** p < 0.001.

**Table 2:** Area under the receiver-operating curve for unique bilateral regions and whole-hemispheric measures of mean cortical thickness.

		Discovery Sample (N = 99)		Replication Sample (N = 20)			Cross-Study Statistics (N = 119)
Region of Interest	Hemisphere	AUC	[95% C.I.]	AUC	[95% C.I.]	Cut-Point	AUC/Precision/Accuracy/Sensitivity/Specificity/F1/LR+/LR−
Banks superior temporal sulcus	Right	0.55	[0.44–0.67]	0.41	[0.15–0.67]	2.60	44.5/73.7/45/42.9/51.6/54.2/0.89/1.11
	Left	0.63	[0.52–0.75]	0.59	[0.31–0.86]	2.68	63/68.4/44.2/41.9/50/52/0.73/1.41
Caudal anterior-cingulate cortex	Right	0.60	[0.48–0.71]	0.63	[0.37–0.89]	2.66	44.1/80.7/45/43.4/52.2/56.4/0.91/1.08
	Left	0.47	[0.36–0.59]	0.66	[0.41–0.91]	2.90	39.5/98.2/43.4/43.8/0/60.5/0.63/1.64
Caudal middle frontal gyrus	Right	0.67	[0.57–0.78]	0.40	[0.13–0.66]	2.71	34/98.2/44.2/44.1/50/60.9/0.88/1.12
	Left	0.59	[0.47–0.70]	0.55	[0.26–0.83]	2.59	43.5/96.5/43.4/43.7/33.3/60.1/1.02/0.98
Cuneus cortex	Right	0.67	[0.56–0.78]	0.44	[0.16–0.71]	2.16	35.2/96.5/44.2/44/50/60.4/0.88/1.12
	Left	0.67	[0.56–0.77]	0.68	[0.43–0.93]	2.05	60/66.7/42.6/40.9/47.2/50.7/1.11/0.93
Entorhinal cortex	Right	0.50	[0.38–0.61]	0.60	[0.33–0.87]	3.53	53/68.4/43.4/41.5/48.6/51.7/0.81/1.2
	Left	0.48	[0.36–0.59]	0.28	[0.04–0.52]	3.27	65/75.4/44.2/42.6/50/54.4/0.9/1.1
Fusiform gyrus	Right	0.65	[0.54–0.76]	0.65	[0.39–0.91]	2.85	40/100/45/44.5/100/61.6/44.53/0.55
	Left	0.66	[0.55–0.77]	0.62	[0.36–0.88]	2.85	55/75.4/44.2/42.6/50/54.4/0.65/1.69
Inferior parietal cortex	Right	0.65	[0.54–0.76]	0.55	[0.28–0.82]	2.71	38.2/100/45/44.5/100/61.6/44.53/0.55
	Left	0.62	[0.51–0.73]	0.68	[0.41–0.95]	2.22	68.5/29.8/50.4/41.5/54.5/34.7/1.03/0.98
Inferior temporal gyrus	Right	0.63	[0.52–0.74]	0.69	[0.42–0.95]	2.58	42.5/29.8/51.2/42.5/55.1/35.1/0.95/1.04
	Left	0.61	[0.50–0.72]	0.62	[0.36–0.89]	2.48	65/5.3/49.6/21.4/53/8.5/0.63/1.3
Isthmus-cingulate cortex	Right	0.59	[0.48–0.70]	0.65	[0.37–0.93]	2.12	45.2/5.3/50.4/23.1/53.4/8.6/0.5/1.44
	Left	0.60	[0.48–0.71]	0.61	[0.33–0.89]	2.85	72/12.3/53.5/41.2/55.4/18.9/1.33/0.83
Lateral occipital cortex	Right	0.66	[0.55–0.77]	0.72	[0.44–1.00]	2.03	40.2/8.8/55/45.5/55.9/14.7/1.03/0.98
	Left	0.71	[0.60–0.81]	0.75	[0.52–0.99]	1.99	54/0/55/0/55.5/0/0.43/1.49
Lateral orbital frontal cortex	Right	0.61	[0.50–0.72]	0.54	[0.27–0.81]	2.84	41.1/98.2/45/44.4/66.7/61.2/1.33/0.83
	Left	0.58	[0.46–0.69]	0.64	[0.38–0.90]	2.40	61.5/0/55/0/55.5/0/0.73/1.23
Lingual gyrus	Right	0.65	[0.55–0.76]	0.62	[0.35–0.88]	2.28	38.9/100/45.7/44.9/100/62/44.88/0.55
	Left	0.63	[0.52–0.74]	0.43	[0.16–0.70]	2.16	62/5.3/53.5/33.3/55/9.1/1.11/0.93
Medial orbital frontal cortex	Right	0.63	[0.52–0.74]	0.62	[0.36–0.88]	1.89	41.9/0/0/0/55.8/0/0/1.79
	Left	0.62	[0.51–0.73]	0.67	[0.42–0.92]	2.74	55/98.2/44.2/44.1/50/60.9/44.53/0.55
Middle temporal gyrus	Right	0.65	[0.54–0.76]	0.55	[0.27–0.83]	2.96	38.9/98.2/45/44.4/66.7/61.2/1.33/0.83
	Left	0.64	[0.53–0.75]	0.67	[0.41–0.93]	3.01	53/96.5/42.6/43.3/0/59.8/0.76/1.32
Parahippocampal gyrus	Right	0.56	[0.45–0.68]	0.53	[0.25–0.81]	3.02	45.1/96.5/43.4/43.7/33.3/60.1/0.65/1.69
	Left	0.51	[0.40–0.63]	0.41	[0.14–0.68]	2.44	37/35.1/48.1/40/53.2/37.4/0.89/1.09
Paracentral lobule	Right	0.57	[0.45–0.68]	0.37	[0.11–0.63]	2.51	42.3/86/45.7/44.1/55.6/58.3/0.99/1.01
	Left	0.62	[0.51–0.74]	0.37	[0.11–0.63]	2.59	44.5/8.8/54.3/41.7/55.6/14.5/44.88/0.55
Pars opercularis	Right	0.61	[0.50–0.72]	0.44	[0.16–0.73]	2.68	41.2/93/46.5/44.9/63.6/60.6/1.24/0.87
	Left	0.66	[0.55–0.77]	0.49	[0.21–0.77]	2.19	50.5/1.8/53.5/20/54.8/3.2/0/1.79
Pars orbitalis	Right	0.55	[0.44–0.67]	0.51	[0.24–0.77]	2.29	45.7/1.8/54.3/25/55.2/3.3/0.56/1.36
	Left	0.44	[0.32–0.55]	0.45	[0.18–0.72]	2.71	57/80.7/43.4/42.6/47.6/55.8/0.66/1.45
Pars triangularis	Right	0.57	[0.46–0.69]	0.57	[0.30–0.84]	2.08	45.3/0/55/0/55.5/0/0/1.8
	Left	0.58	[0.47–0.70]	0.34	[0.08–0.60]	2.03	35/7/51.9/30.8/54.3/11.4/2.29/0
Pericalcarine cortex	Right	0.62	[0.51–0.73]	0.35	[0.09–0.61]	1.38	37.7/7/53.5/36.4/55.1/11.8/0.81/1.16
	Left	0.62	[0.51–0.73]	0.51	[0.23–0.79]	1.30	74/49.1/48.1/42.4/54/45.5/2.29/0
Postcentral gyrus	Right	0.63	[0.52–0.74]	0.74	[0.50–0.98]	2.30	43/100/45.7/44.9/100/62/44.88/0.55
	Left	0.67	[0.56–0.77]	0.59	[0.32–0.86]	2.36	59/98.2/43.4/43.8/0/60.5/44.53/0.55
Posterior-cingulate cortex	Right	0.60	[0.49–0.71]	0.59	[0.33–0.85]	2.71	43.2/98.2/44.2/44.1/50/60.9/0.88/1.12
	Left	0.57	[0.45–0.68]	0.50	[0.22–0.77]	2.27	41.5/98.2/43.4/43.8/0/60.5/0.54/1.39
Precentral gyrus	Right	0.67	[0.57–0.78]	0.42	[0.15–0.68]	2.74	34.5/100/45.7/44.9/100/62/44.88/0.55
	Left	0.72	[0.62–0.82]	0.36	[0.09–0.63]	2.77	52/98.2/43.4/43.8/0/60.5/44.53/0.55
Precuneus cortex	Right	0.66	[0.55–0.76]	0.52	[0.25–0.79]	2.83	37.8/100/45/44.5/100/61.6/44.53/0.55
	Left	0.64	[0.53–0.75]	0.53	[0.26–0.80]	2.54	64/8.8/48.8/26.3/52.7/13.2/1.79/0.74
Rostral anterior cingulate cortex	Right	0.53	[0.41–0.64]	0.64	[0.37–0.91]	2.43	49.9/8.8/50.4/29.4/53.6/13.5/0.63/1.32
	Left	0.59	[0.48–0.71]	0.78	[0.55–1.00]	2.04	57/98.2/43.4/43.8/0/60.5/0/1.79
Rostral middle frontal gyrus	Right	0.63	[0.52–0.74]	0.57	[0.30–0.84]	2.49	40.6/98.2/44.2/44.1/50/60.9/0.88/1.12
	Left	0.69	[0.59–0.79]	0.65	[0.39–0.91]	2.03	48/98.2/43.4/43.8/0/60.5/0/1.79
Superior frontal gyrus	Right	0.67	[0.56–0.77]	0.48	[0.21–0.75]	2.91	35.3/100/45/44.5/100/61.6/44.53/0.55
	Left	0.63	[0.52–0.74]	0.43	[0.15–0.71]	2.89	55/98.2/43.4/43.8/0/60.5/44.53/0.55
Superior parietal cortex	Right	0.67	[0.57–0.78]	0.55	[0.26–0.84]	2.50	37.1/100/45/44.5/100/61.6/44.53/0.55
	Left	0.61	[0.49–0.72]	0.62	[0.36–0.88]	2.36	51/5.3/55/42.9/55.7/9.4/0.76/1.32
Superior temporal gyrus	Right	0.68	[0.57–0.78]	0.51	[0.24–0.78]	3.15	36.5/100/45/44.5/100/61.6/44.53/0.55
	Left	0.66	[0.55–0.77]	0.42	[0.15–0.69]	2.99	57/98.2/44.2/44.1/50/60.9/44.88/0.55
Supramarginal gyrus	Right	0.71	[0.61–0.82]	0.57	[0.29–0.85]	2.77	33.2/98.2/45/44.4/66.7/61.2/1.33/0.83
	Left	0.66	[0.55–0.76]	0.59	[0.32–0.86]	2.86	44.5/0/54.3/0/55.1/0/44.53/0.55
Frontal pole	Right	0.63	[0.52–0.74]	0.45	[0.16–0.73]	2.10	38.9/0/55/0/55.5/0/0/1.8
	Left	0.67	[0.56–0.77]	0.31	[0.05–0.56]	3.50	33.1/100/45/44.5/100/61.6/44.53/0.55
Temporal pole	Right	0.56	[0.44–0.67]	0.37	[0.10–0.64]	2.99	44.3/7/55/44.4/55.8/12.1/1.01/1
	Left	0.57	[0.46–0.69]	0.49	[0.21–0.77]	3.87	52/87.7/43.4/43.1/46.2/57.8/0.87/1.13
Transverse temporal cortex	Right	0.66	[0.55–0.78]	0.52	[0.24–0.80]	2.52	37.4/82.5/45/43.5/52.4/57/0.91/1.08
	Left	0.65	[0.54–0.76]	0.58	[0.31–0.86]	2.66	50/87.7/45/43.9/53.3/58.5/44.53/0.55
Insula	Right	0.64	[0.53–0.76]	0.50	[0.23–0.77]	3.12	38.4/87.7/45.7/44.2/56.3/58.8/1.01/0.99
	Left	0.68	[0.57–0.79]	0.52	[0.24–0.80]	3.21	50/87.7/45/43.9/53.3/58.5/1.58/0.77
Mean Hemispheric Cortical Thickness	Right	0.71	[0.61–0.81]	0.61	[0.34–0.88]	2.65	41/70.2/44.2/42.1/50/52.6/44.53/0.55 50/87.7/45/43.9/53.3/58.5/44.53/0.55
	Left	0.70	[0.60–0.81]	0.58	[0.31–0.85]	2.67	41/70.2/44.2/42.1/50/52.6/44.53/0.55

**Table 3:** Marginal signal intensity as reported by the artificial neural network relying on 68 unilateral regions of interest and reported in the discovery sample.

Region	Hemisphere	Signal Margins
Banks superior temporal sulcus	Right	‒0.011
	Left	0.181
Caudal anterior-cingulate	Right	‒0.154
	Left	0.226
Caudal middle frontal	Right	‒0.273
	Left	0.335
Cuneus	Right	‒0.313
	Left	0.168
Entorhinal	Right	0.233
	Left	0.156
Frontal pole	Right	‒0.217
	Left	‒0.042
Fusiform	Right	0.126
	Left	0.185
Inferior parietal	Right	‒0.048
	Left	0.024
Inferior temporal	Right	‒0.230
	Left	‒0.002
Isthmus-cingulate	Right	0.000
	Left	‒0.174
Lateral occipital	Right	0.268
	Left	‒0.009
Lateral orbital frontal	Right	‒0.122
	Left	0.060
Lingual	Right	‒0.001
	Left	0.034
Medial orbital frontal	Right	0.007
	Left	0.002
Middle temporal	Right	‒0.388
	Left	‒0.192
Paracentral lobule	Right	0.013
	Left	0.035
Parahippocampal	Right	0.346
	Left	‒0.005
Pars opercularis	Right	0.239
	Left	‒0.224
Pars orbitalis	Right	‒0.001
	Left	0.272
Pars triangularis	Right	0.184
	Left	‒0.115
Pericalcarine	Right	‒0.193
	Left	‒0.123
Postcentral	Right	0.210
	Left	‒0.079
Posterior-cingulate	Right	0.104
	Left	‒0.163
Precentral	Right	‒0.192
	Left	‒0.440
Precuneus	Right	0.100
	Left	‒0.166
Rostral anterior cingulate	Right	‒0.163
	Left	‒0.026
Rostral middle frontal	Right	0.163
	Left	‒0.263
Superior frontal	Right	‒0.413
	Left	0.174
Superior parietal	Right	0.000
	Left	0.103
Superior temporal	Right	‒0.009
	Left	‒0.042
Supramarginal	Right	‒0.127
	Left	0.158
Temporal pole	Right	0.001
	Left	0.011
Transverse temporal	Right	‒0.006
	Left	‒0.253
Insula	Right	0.146
	Left	‒0.120
Constant		0.000
