# Supplementary material for: A cortical thinning signature to identify World Trade Center responders with possible dementia
Source: Intell Based Med. Author manuscript; Available in PMC 2022 Aug 18. (PMC9387912; doi:10.1016/j.ibmed.2021.100032)
Supplement: Appendix Tables and Figures [file NIHMS1775482-supplement-Appendix_Tables_and_Figures.docx]

**Supplementary Figure 1. The learning curve for the Artificial Neural Network (ANN) algorithm.** Shown are the predictive accuracies, including the mean accuracy in the randomly selected training subsample (black dashed line) and the mean accuracy of the randomly selected testing subsample (red dashed line; 95% confidence intervals are shown by the thin gray lines).

**Supplementary Table 1. Cognitive Measurements used in the present study.** Measures of cognition provided by the CogState Computer-assisted cognitive measurement tool

| Cognitive Domain | Units | Direction | Task Used |
| --- | --- | --- | --- |
| Response speed | Responses/Second | Higher is better | Detection |
| Processing speed | Correct Responses/Second | Higher is better | Identification |
| intra-item response variability | Standard Deviations | Higher is worse | Identification |
| Attention | arcsine-Correct responses | Higher is better | Identification |
| Visual memory | arcsine-Correct responses | Higher is better | One-card learning |
| Throughput | arcsine-Correct responses/second | Higher is better | One-card learning |

**Supplementary Table 2**. **Artificial Neural Network signals, marginal estimates, and standardized differences.** Estimates of standardized mean differences are adjusted for unequal group sizes (*g*) for World Trade Center responders identified as at high atrophy risk as compared to those in the low atrophy risk group.

| CogState Variable | Margins | Signal | Hedge's g |
| --- | --- | --- | --- |
| Intra-individual response variability | 0.222 | 0.010 | 1.952 |
| Response speed | -0.474 | -0.677 | -1.493 |
| Processing speed | 0.037 | 0.004 | -1.106 |
| Attention | -0.146 | -0.033 | -0.196 |
| Visual memory | 0.207 | 0.010 | -0.797 |
| Throughput | -0.693 | -0.990 | -1.103 |
